# Supplementary material for: Scorpion Peptide Smp24 Exhibits a Potent Antitumor Effect on Human Lung Cancer Cells by Damaging the Membrane and Cytoskeleton In Vivo and In Vitro
Source: Toxins (Basel). 2022 Jun 28;14(7):438. doi: 10.3390/toxins14070438 (PMC9318729; doi:10.3390/toxins14070438)
Supplement: Supplementary file 1 [file toxins-14-00438-s001.zip › toxins-1773079-supplementary.pdf]

Supplementary Materials: Scorpion Peptide Smp24 Exhibits a Potent Antitumor Effect on Human Lung Cancer Cells by Damaging the Membrane and Cytoskeleton In Vivo and In Vitro

Ruiyin Guo, Junfang Liu, Jinwei Chai, Yahua Gao, Mohamed A. Abdel–Rahman and Xueqing Xu

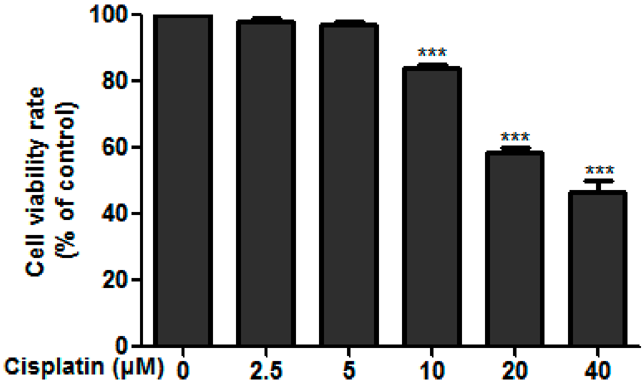

Figure S1. Viability of A549 cells treated with cisplatin for 24 h.

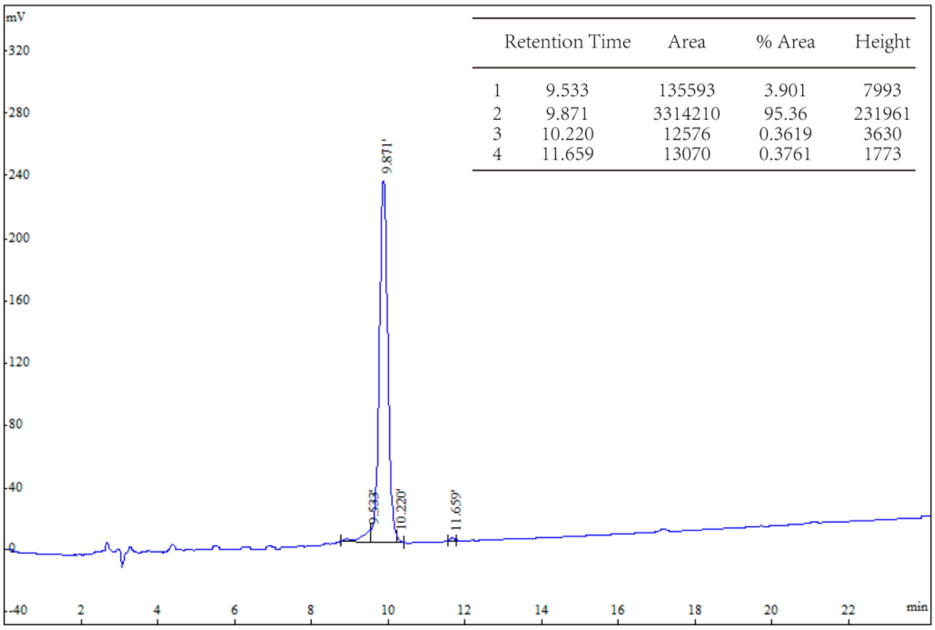

	Retention Time	Area	% Area	Height
1	9.533	135593	3.901	7993
2	9.871	3314210	95.36	231961
3	10.220	12576	0.3619	3630
4	11.659	13070	0.3761	1773

Figure S2. RP-HPLC purification of Smp24.

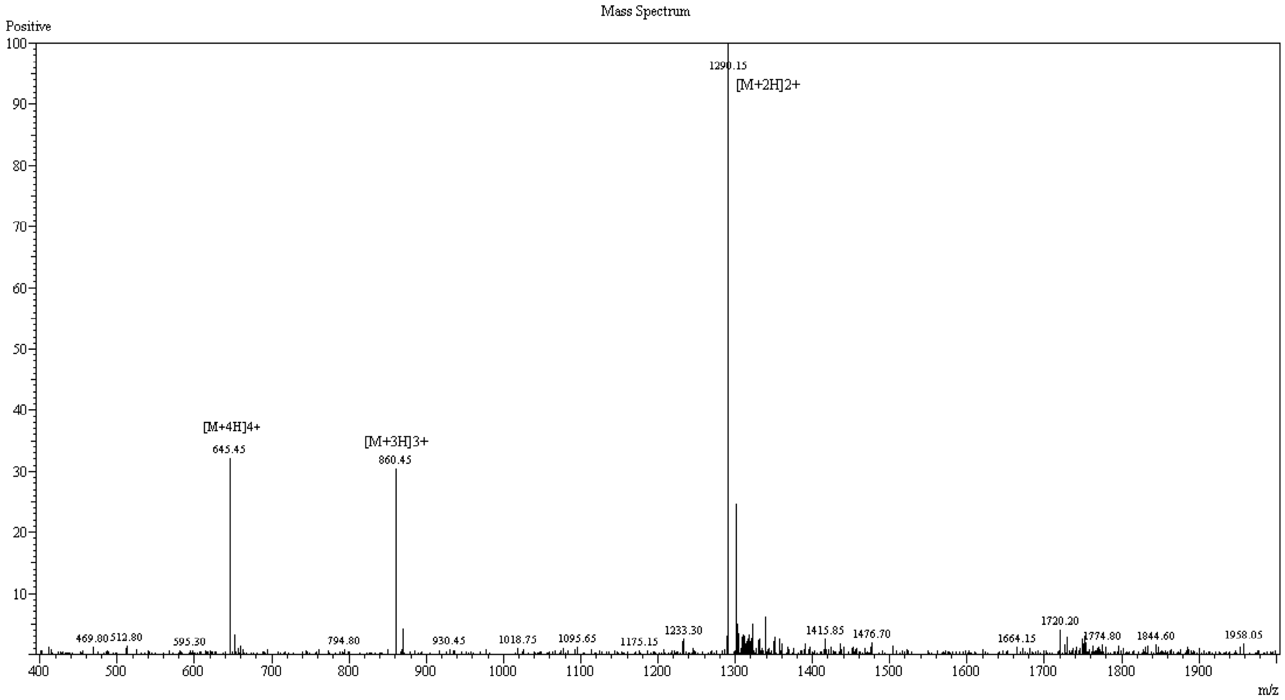

Figure S3. MALDI-TOF-MS identification of Smp24.

Table S1. IC_{50} values and selectivity index of cancer cell lines treated with Smp24 for 24 h.

	**IC_{50} (μM)**	**Selectivity Index**
A549	4.06 ± 0.60	3.61
H3122	4.64 ± 0.18	3.16
PC-9	6.34 ± 0.53	2.32
H460	7.07 ± 0.81	2.07
MRC-5	14.68 ± 0.79	-
